# Editorial: Non-cutaneous melanoma: new therapeutic insights

**DOI:** 10.3389/fonc.2023.1362238

**Published:** 2024-01-17

**Authors:** Ernesto Rossi, Richard D. Carvajal

**Affiliations:** ^1^Medical Oncology, Fondazione Policlinico Universitario Agostino Gemelli IRCCS, Rome, Italy; ^2^Hematology and Medical Oncology, Northwell Health Cancer Institute, Lake Success, NY, United States

**Keywords:** uveal melanoma, mucosal melanoma, extracutaneous melanoma, melanoma treatment, non-cutaneous melanoma

Extraordinary advances have been made in recent years for the treatment of melanoma, with a number of effective new drugs receiving regulatory approval; however, these achievements have predominantly impacted patients with cutaneous melanoma. Extracutaneous melanomas, including uveal and mucosal melanomas, are clinically and biologically distinct from melanoma of the skin, less frequent in terms of incidence compared to cutaneous disease, are less commonly the focus of large-scale research efforts, and are associated with inferior clinical outcomes. Uveal melanoma is a rare neoplasm, but is the most common primary malignancy of the eye, occurring in about 5.2 patients/million/year (1, 2). Mucosal melanoma arises from mucosal tissue of head and neck (eg. oral cavity and sinonasal mucosa), gastrointestinal tract or urogenital tract, and comprises less than 1% of all melanoma diagnoses (3). Despite the recent approval of tebentafusp for HLA-A*02:01 positive patients with advanced uveal melanoma (4–7) and the results with HEPZATO KIT as a liver-directed treatment for patients with unresectable uveal melanoma hepatic metastases affecting less than 50% of the liver and no extrahepatic disease, or extrahepatic disease limited to the bone, lymph nodes, subcutaneous tissues, or lung that is amenable to resection or radiation (8), outcomes remain poor for these patient populations. This Research Topic was specifically dedicated to research focused on extracutaneous melanomas in an effort to increase awareness, strengthen the rare melanoma research community, and improve the outcomes for patients with these diseases.

Despite the strength of national and regional cancer registries which provide population-based cancer incidence data, the ability of these registries to provide robust data on rare melanomas is more limited. The accuracy and completeness of registry data is reliant upon the chart reviews performed by hospital registrars. While the diagnosis of most cancers relies upon confirmatory pathology or radiologic imaging, the diagnosis of uveal melanoma is based on clinical features and characteristic findings on examination, making it challenging for hospital registrars to identify and report these cases using their typical workflows. In this Research Topic, Gordon et al. reports on the impact of the unique diagnostic course of uveal melanoma at one institution and the challenges this posed to case reporting to the state cancer registry, which affect our estimates of true disease incidence and prevalence.

Within this Research Topic, we also include studies providing novel insights into the pathogenesis and natural history of uveal melanoma. Increasing evidence highlights the role of DNA methylation in the development of ocular melanoma. Yang et al. conducted a study to explore the correlation between DLL3 (delta-like-protein 3) and the methylation of its corresponding CpGs in uveal melanoma and observed that the methylation levels of CpGs located in DLL3 gene are correlated with DDL3 expression. High DLL3 expression was associated with prolonged overall survival and disease-free survival, nominating DLL3 as a prognostic factor in uveal melanoma. The hepatotropism of uveal melanoma metastases has been well documented, with spread to the brain considered to be uncommon. Wei et al. conducted a large multicenter retrospective analysis which identified brain metastases in 6% of patients with uveal melanoma, with the presence of brain involvement associated with high disease burden. These data suggest that brain imaging should be considered in patients with metastases involving multiple organs and who have had disease progression on multiple lines of therapy.

Given the high risk for development of metastatic recurrence despite treatment of the primary tumor (9), the identification of an effective adjuvant therapy is a critical need within the uveal melanoma field; however, the conduct of definitive randomized trials is challenging due to the rarity of this disease as well as the trial costs. Khan et al. report on the results of a single arm, multicenter phase 2 trial of crizotinib, a multitargeted tyrosine kinase inhibitor targeting cMET, which is highly expressed in the majority of uveal melanomas, in patients with uveal melanoma at high-risk of disease recurrence following treatment of the primary tumor. Using an external data set to develop a synthetic control with propensity score methods, adjuvant crizotinib was demonstrated to not achieve improved outcomes when compared to historical controls.

Preclinical data has demonstrated the dependency of uveal melanoma upon MAPK signaling due to constitutive activation of the G alpha pathway, leading to the conduct of multiple trials evaluating the efficacy of continuous dosing of MEK1/2 inhibition alone with selumetinib and other MEK inhibitors, without meaningful efficacy observed (10, 11). As intermittent MEK inhibition reduces compensatory pathway activation and induces cytotoxic T cell function, Khan et al. performed a phase Ib dose escalation study of selumetinib administered on a 3 day on/4 day off schedule. Although the maximum tolerated dose utilizing this schedule was double that identified when using a continual dosing schedule, with MAPK pathway inhibition observed at day 3 using tumor biopsies, no significant benefit was observed. Protein kinase C (PKC) is also downstream of the G alpha pathway and has been implicated in the pathogenesis of uveal melanoma, with synergistic antitumor activity observed with concurrent inhibition of PKC and MEK (12). Bauer et al. conducted a phase Ib study with sotrastaurin, a potent, oral, selective inhibitor of the classical and novel PKC isoforms (13), and binimetinib, a MEK inhibitor, in patients with advanced uveal melanoma. Although two dose combinations satisfying criteria for the maximum tolerated dose were identified, inadequate target inhibition was achieved with the tolerable doses administered and limited activity was observed.

Despite the negative results of the three uveal melanoma clinical trials, progress is being made in the field (14). Indeed, darovasertib, a next generation PKC inhibitor that is more potent in inhibiting both conventional and novel PKC isoforms with better tolerability than sotrastaurin, has demonstrated activity alone and in combination with both binimetinib and crizotinib in uveal melanoma (15, 16). The trials of darovasertib alone in the neoadjuvant setting (NCT05907954) and the combination of darovasertib and crizotinib in first-line HLA-A2-negative metastatic uveal melanoma (NCT05987332) are now accruing. In November 2023, the European Organisation for Research and Treatment of Cancer (EORTC) announced a collaboration with Immunocore to develop and launch the ATOM trial investigating the adjuvant treatment of uveal melanoma with tebentafusp in HLA-A*02:01 positive patients with high-risk disease following treatment of the primary lesion.

As in uveal melanoma, the efficacy of the clinically available immune checkpoint inhibitors is lower in mucosal melanoma than in cutaneous disease (17); however, up to 37% of patients with advanced mucosal melanoma may respond to the combination of anti-PD1 and anti-CTLA4 therapy (18). Furthermore, while the efficacy of neoadjuvant checkpoint inhibition has been evaluated in a large, randomized trial in cutaneous melanoma, only limited data are available regarding neoadjuvant therapy in mucosal disease. In this Research Topic, Ho et al. assess the clinical and pathological response rates observed with neoadjuvant combined checkpoint blockade in mucosal melanoma, and report an overall radiological response rate of 47% and a complete or partial pathologic response rate of 36%, with an improved overall survival associated with pathologic response.

Several randomized trials have now demonstrated the efficacy of adjuvant chemotherapy over interferon-α2b or observation in patients with resected mucosal melanoma (19). Tang et al. explored the proliferation marker Ki67 as predictive biomarker of efficacy for adjuvant chemotherapy in resectable mucosal melanoma in 175 patients treated with adjuvant temozolomide based-chemotherapy or high dose interferon-α2b. For patients with tumors characterized by high Ki67 expression (≥30%), temozolomide based-adjuvant therapy achieved a RFS of 18 months compared to 6.7 months with interferon (p<0.001); however, no difference in outcomes was observed in those with tumors having lower proliferation.

Moving forward, our hope is that continued efforts such as the collaboration that resulted in this Research Topic will increase support of further studies and international work focused on extracutaneous melanoma. Enhancing cooperation is critical for us to more rapidly increase knowledge in the field and improve the outcomes for our patients with these rare diseases.

## Author contributions

ER: Writing – original draft, Writing – review & editing. RC: Writing – original draft, Writing – review & editing.
